# Recommended dosages of analgesic and sedative drugs in intensive care result in a low incidence of potentially toxic blood concentrations

**DOI:** 10.48101/ujms.v129.10560

**Published:** 2024-05-09

**Authors:** Ulrica Lennborn, Anna Johansson, Erik Lindgren, Elisabet I. Nielsen, Håkan Sandler, Maria Bertilsson, Robert Kronstrand, Johan Ahlner, Fredrik C. Kugelberg, Sten Rubertsson

**Affiliations:** a Department of Surgical Sciences, Division of Anaesthesiology and Intensive Care Medicine, Uppsala University, Uppsala, Sweden; b Department of Forensic Genetics and Forensic Toxicology, National Board of Forensic Medicine, Linköping, Sweden; c Department of Pharmacy, Uppsala University, Uppsala, Sweden; d Department of Surgical Sciences/Forensic Medicine, Uppsala University, Uppsala, Sweden; e Uppsala Clinical Research Centre, Uppsala University, Uppsala Sweden; f Department of Biomedical and Clinical Sciences, Division of Clinical Chemistry and Pharmacology, Linköping University, Linköping, Sweden

**Keywords:** Analgesia, sedation, drug dosages, drug concentrations, intensive care medicine, critical care

## Abstract

**Background:**

Standard dosages of analgesic and sedative drugs are given to intensive care patients. The resulting range of blood concentrations and corresponding clinical responses need to be better examined. The purpose of this study was to describe daily dosages, measured blood concentrations, and clinical responses in critically ill patients. The purpose was also to contribute to establishing whole blood concentration reference values of the drugs investigated.

**Methods:**

A descriptive study of prospectively collected data from 302 admissions to a general intensive care unit (ICU) at a university hospital. Ten drugs (clonidine, fentanyl, morphine, dexmedetomidine, ketamine, ketobemidone, midazolam, paracetamol, propofol, and thiopental) were investigated, and daily dosages recorded. Blood samples were collected twice daily, and drug concentrations were measured. Clinical responses were registered using Richmond agitation-sedation scale (RASS) and Numeric rating scale (NRS).

**Results:**

Drug dosages were within recommended dose ranges. Blood concentrations for all 10 drugs showed a wide variation within the cohort, but only 3% were above therapeutic interval where clonidine (57 of 122) and midazolam (38 of 122) dominated. RASS and NRS were not correlated to drug concentrations.

**Conclusion:**

Using recommended dose intervals for analgesic and sedative drugs in the ICU setting combined with regular monitoring of clinical responses such as RASS and NRS leads to 97% of concentrations being below the upper limit in the therapeutic interval. This study contributes to whole blood drug concentration reference values regarding these 10 drugs.

## Introduction

Most patients in the intensive care unit (ICU) are treated with a variety of sedative and analgesic drugs. It is not fully clear how best to determine dosage regimens of analgesics and sedatives in the clinical care of critically ill patients (1). Limited data exist about the pharmacokinetics in ICU patients of some of the most used analgesic and sedative drugs when given for more than 24 h in a real-world setting. Pharmacokinetic-pharmacodynamic (PK/PD) relationships are complex, and PK/PD models for optimized drug delivery in the ICU are not yet fully established (2–4). Previous population pharmacodynamic models show a wide interindividual variability in the pharmacodynamics of the sedatives dexmedetomidine, midazolam, and propofol (5–7). Other studies show that analgesic and sedative drug pharmacokinetics during critical illness are influenced by severe liver disease, kidney failure as well as age and weight (8, 9), and the drug concentrations achieved with standard dosages administered to an ICU population can vary significantly between patients (10, 11). Long-term use and several different combinations of analgesic and sedative drugs may lead to adverse effects, for example, over-sedation and extended ICU length of stay (12, 13).

Historically, clinical studies have used plasma to determine drug concentrations, and therefore, almost all reference values are from plasma (14). In the field of postmortem toxicology on the other hand, whole blood has been the sample material of choice. This could be a result of plasma being difficult to generate from a deceased person, as the blood cells could have hemolyzed. Plasma concentrations are not equivalent to whole blood concentrations (15). We have analyzed drug concentrations in whole blood instead of the historically used plasma to be able to compare the results from samples obtained from the living to those taken at autopsy.

In this single-center prospective study of a Swedish general ICU population, the aim was to describe the use of 10 sedative and analgesic drugs, including dosages, measured blood concentrations, and clinical responses. A second aim was to contribute to the establishment of whole blood concentration reference values for the 10 drugs investigated.

## Materials and methods

A descriptive study of prospectively collected data was performed in the general ICU at Uppsala University hospital, Sweden. In a 1-month pilot phase preceding the main study, including 69 patients, the study logistics including blood sampling and transportation were tested. No logistic or feasibility issues were found.

### Compliance with ethical standards

This study was approved and an informed consent was waived by the Uppsala Ethics Review Board, Sweden (Reg. no. 2012/559). This study was conducted in accordance with regulatory requirements, and the ethical principles of the Declaration of Helsinki (16).

### Selection and description of participants

**Figure 1:**
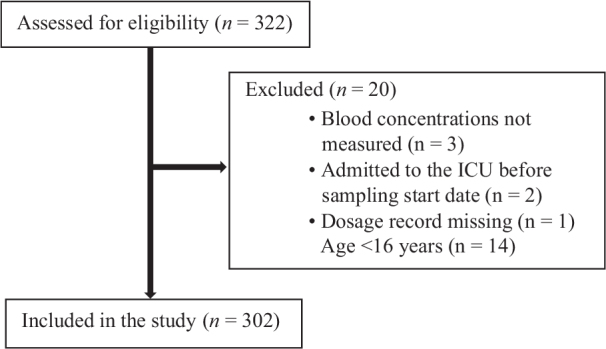
Flowchart of inclusion and exclusion (n = ICU admissions).

We included all patients from age 16, admitted to a Swedish general ICU. This study was performed with a duration of 5 months in 2015 (Figure 1).

### Data collection

Age, sex, medical history, and diagnosis at discharge were recorded. The Simplified Acute Physiology Score (SAPS) 3 and Expected Mortality Rate (EMR) were calculated (17, 18). The patients’ reasons for treatment in the ICU were divided into 12 diagnose groups according to the diagnosis given at discharge from the ICU.

### Sampling procedures

Blood sampling was performed according to routine procedure upon the patient’s arrival at the ICU and then twice daily [at 6 am and 6 pm] until discharge. Whole blood for analysis of sedatives and analgesics was collected in 9 mL Venosafe VF-109SFX07 tubes (Terumo Europe NV, Leuven, Belgium) containing 100 mg of sodium fluoride and 22.5 mg of potassium oxalate. All samples were stored in a refrigerator at 5°C until transportation to the National Board of Forensic Medicine in Linköping, Sweden. During the transport, blood samples were stored in refrigerated boxes. Upon arrival, the samples were unpacked, registered, and then immediately refrigerated at 4°C. Routine blood samples were analyzed at the central laboratory in Uppsala University Hospital.

### Chemical analysis

This study focused on 10 sedative and analgesic drugs: dexmedetomidine, midazolam, propofol, ketamine, clonidine, thiopental, fentanyl, ketobemidone, morphine, and paracetamol. However, all admission samples were screened for 224 drugs and metabolites, using liquid chromatography high-resolution mass spectrometry, to identify drugs taken by the patient outside of the hospital and to examine if any of the 10 focused drugs were present before given in the hospital (19).

Dexmedetomidine, midazolam, ketamine, clonidine, fentanyl, ketobemidone, and morphine were quantified by means of a liquid chromatography tandem mass spectrometry method developed and validated for this study (20). The calibration ranges were 0.00025–0.050 µg/g for dexmedetomidine, 0.05–50 ng/g for fentanyl, 0.005–0.500 µg/g for morphine, ketobemidone, and midazolam, 0.0005–0.050 µg/g for clonidine, and 0.05–5.0 µg/g for ketamine. Dilution integrity was verified, and results above the calibration range were diluted.

The remaining drugs were analyzed by routine methods at the National Board of Forensic Medicine. Paracetamol was quantified using liquid chromatography tandem mass spectrometry with a calibration range of 1–100 µg/g. Propofol was quantified using gas chromatography mass spectrometry with a calibration range of 0.05–3.0 µg/g. Thiopental was quantified using gas chromatography nitrogen phosphorous detection with a calibration range of 0.5–20 µg/g.

### Drug dosage monitoring and concentration measurement

Drugs were given according to standard dosing regimens for analgesia and sedation (Table 1) (21). Administered drug dosages, including bolus dosages and changes of infusion rates, were documented. During a 24-h interval between 5 am and 5 am, according to the routines in this ICU, the total given amount of each drug was registered as a 24-h dosage, and then at 6 am, the day’s first blood sample was drawn.

**Table 1:** Standard dose regimens for sedatives and analgesics in the ICU.

Drug substance	Recommended starting dose	Dose interval	Max dose	Unit	Reference 24 h dose (min-max, 75 kg person)
Clonidine	0,125	0.1–0.5	1	ug/kg/h	180–1800 ug
Dexmedetomidine	0,2	0.2–1.4	2	ug/kg/h	360–3600 ug
Fentanyl	0,5	0.5–1.8	3	ug/kg/h	900–5400 ug
Ketamine	1	0.25–3	3	mg/kg/h	450–5400 mg
Ketobemidone	10	10–100	100	ug/kg/h	18–180 mg
Midazolam	0,03	0.03–0.2	0.2	mg/kg/h	54–360 mg
Morphine	10	10–100	100	ug/kg/h	18–180 mg
Paracetamol	-	0.5–4	4	g/d	0.5–4 g
Propofol	2	0.5–4	4	mg/kg/h	900–7200 mg
Thiopental	3	1–4	6	mg/kg/h	1800–10800 mg

Samples for drug concentration analysis were taken twice daily, from the first day the drug was given until the measured concentration was below the limit of quantification, or until discharge from the ICU. We here present all analyzed concentrations, including days when the dosage was zero (0), that is, no ongoing active treatment.

### Registration of clinical response

Sedation level was evaluated by the Richmond Agitation Scale (RASS) (22). RASS was recorded three times daily for patients with ongoing sedation. The local recommended sedation level was RASS–2. An assessment of the Numeric rating scale (NRS) was done three times daily in patients who were awake and able to cooperate (23).

Concentrations for four of the most common drugs (propofol, dexmedetomidine, fentanyl, and morphine) were related to RASS level or NRS rate registered within 4 h of blood sampling time.

### Statistical analysis

Due to the observational and exploratory nature of this study, only descriptive statistics and graphical presentations are used. Baseline characteristics, drug dosages, and concentrations were summarized using medians, interquartile ranges, and minimum and maximum for continuous variables, and using counts and percentages for categorical variables. The association between propofol and RASS was visualized using scatter plots with different colors depicting the presence of concomitant medication with fentanyl or dexmedetomidine. Associations among analgesic drug concentrations, fentanyl or morphine, and NRS were presented as scatter plots. All analyses were performed using SAS (version 9.4, SAS Institute, Cary, North Carolina, USA).

## Results

### Background data

Of the 302 admissions, 21 individuals (7%) were re-admitted to the ICU but were included in the demographic data only once. We chose to include a patient as a new admission if they had been away from the ICU more than 24 h. In the 281 patients included in our demographic data, median age was 63 years, and 58% were male. The most common diagnosis at discharge from the ICU was postoperative intensive care (30%). The median ICU length of stay was 1.1 days, and the mean was 2.7 days (Table 2).

**Table 2:** Population demographics upon admission for the first ICU visit (*n* = 281*).

Variable	Count = *n* (%)	Median	Mean	Min	Max
Female	119 (42)				
Male	162 (58)				
Age	280	63		16	91
Weight	281	83		34	146
SAPS 3	280	55		20	105
EMR	280**	13.5		0.1	89.3
RASS	4081	-2		-5	3
NRS	407	4		0	8
ICU days	281	1.1	2.7	0.04	66.7
Diagnosis at discharge: Postoperative Infectious Pulmonary Cardiovascular Intoxication Trauma Neurology Nephrology Gastroenterology Endocrinology Oncology Burn injury	*n* (%) 83 (30) 39 (14) 34 (12) 29 (10) 23 (8) 21 (7) 11 (4) 13 (5) 11 (4) 9 (3) 5 (2) 3 (1)				
Deceased	22 (8)				

* We included 302 ICU admissions in the study. Of these, 21 patients were re-admitted up to four times to the ICU, and, therefore, demographic data are presented only once. This resulted in demographic data from 281 individual patients.

** One SAPS3 value was missing and could therefore not generate an EMR.

### Drug dosages

All daily dosages were within recommended range of doses in relation to the individual patient’s weight (Table 3). Ninety patients (30%) received 333 single doses (15%) of analgesic and sedative drugs in the operating room, either right before admission to the ICU or sometime during the ICU stay. These doses were included in the 24-h dosages and in the tables and graphs. Propofol, given to 50% of the patients, and fentanyl (56%) were the most commonly used sedative and analgesic drugs, in accordance with the recommended sedation regimens at the time (24). Among patients sedated with propofol, 72% were also given fentanyl and 17% dexmedetomidine.

**Table 3:** Daily drug dosages given per substance during the entire stay in the ICU.

Drug substance	24h doses (*n*)	Median	Min	Max	Q1	Q3	Unit
Clonidine	205	300	4.7	2632.5	135	570	ug
Dexmedetomidine	177	604.5	1	4015.8	160	1364.8	ug
Fentanyl	502	650	25	8300	288	1750	ug
Ketamine	100	500	8	4312.8	200	1738.3	mg
Ketobemidone	104	4.25	0.5	50	2	8.5	mg
Midazolam	105	50	0.5	349.3	20	143.7	mg
Morphine	236	10.4	1	190	4.3	28	mg
Paracetamol	283	2	0.5	5	1	4	g
Propofol	546	1376.2	0.3	18500	500	3460	mg
Thiopental	7	1050	200	8750	200	4875	mg

### Concentrations and clinical response

The blood concentrations measured showed a wide variability. The most frequently measured drugs were fentanyl (*n* = 1,008) and propofol (*n* = 907), whereas only 27 measurements were performed for thiopental (Table 4). The blood concentrations measured were above therapeutic plasma concentration reference values in 122 out of 3,827 measurements (3%). Of the concentration values above therapeutic interval, the most frequent were clonidine (57 of 122), midazolam (38 of 122), and paracetamol (11 of 122).

**Table 4:** Descriptive statistics, all measured whole blood concentrations per substance.

Drug substance	Patients (*n*)	Concen-trations (n)	Median	Min	Max	Q1	Q3	Therapeutic plasma concentrations^d^ (min-max)	B/P^e^	Unit^f^
Clonidine	61	347	0.0016	0.0005	0.017	0.001	0.003	0.001– 0.004	0.5	ug/g
Dexmedetomidine	44	209	0.0008	0.0003	0.0073	0.0006	0.0013	Unknown	Unknown	ug/g
Fentanyl^a^	166	1008	0.71	0.05	9.4	0.21	1.3	3–10	0.8–1.0	ng/g
Ketamine	42	174	0.58	0.05	4.5	0.12	1.3	1–6	1.6–1.7	ug/g
Ketobemidone	30	79	0.009	0.005	0.13	0.006	0.013	0.01 – 0.05	Unknown	ug/g
Midazolam	44	222	0.075	0.005	1.2	0.021	0.18	0.04–0.1	0.5–0.7	ug/g
Morphine	113	280	0.016	0.005	0.21	0.009	0.026	0.01–0.1	1.0	ug/g
Paracetamol^b^	137	574	5	1	48	3	9	5–25	1.1	ug/g
Propofol^c^	150	907	0.89	0.05	16.5	0.24	1.7	0.8–8	1–2	ug/g
Thiopental	5	27	2	0.6	28.5	1	15.3	1–5	1.0	ug/g

a One sample excluded [70 ng/g].

b One sample excluded [77 ug/g], voluntary intoxication with paracetamol before admission to ICU.

c Two samples excluded [70 ug/g and >100 ug/g].

d Therapeutic plasma concentrations accordingly to Schulz et al Revisited: Therapeutic and toxic blood concentrations of more than 1100 drugs and other xenobiotics. *Crit Care* **24**, 2020.

e Blood/plasma ratios accordingly to Baselt R. (ed) Disposition of toxic drugs and chemicals in man. 12th ed. (2020) Biomedical publications, Seal Beach, CA, USA.

f Units are per weight (gram) since whole blood aliquots were weighed for analysis. The weight/volume ratio for whole blood is 1.055.

**Figure 2:**
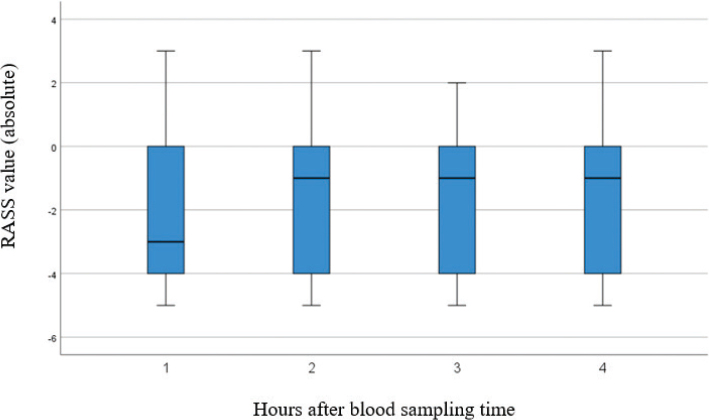
RASS values measured within 1, 2, 3, and 4 h from the sampling time. The marked line is the median RASS value.

**Figure 3:**
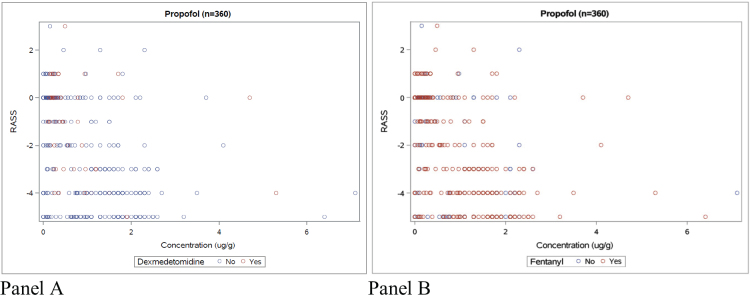
Propofol concentrations with corresponding RASS value within 4 h of sampling time. Panel A: with and without dexmedetomidine. Panel B: with and without fentanyl.

RASS values for the individual patient did not significantly vary between 1, 2, 3, and 4 h from blood sampling time (Figure 2). Sedation levels evaluated by RASS within 4 h from blood sampling were widely spread in relation to corresponding propofol concentrations, regardless of whether another sedative was present or not (Figure 3, panels A and B).

**Figure 4:**
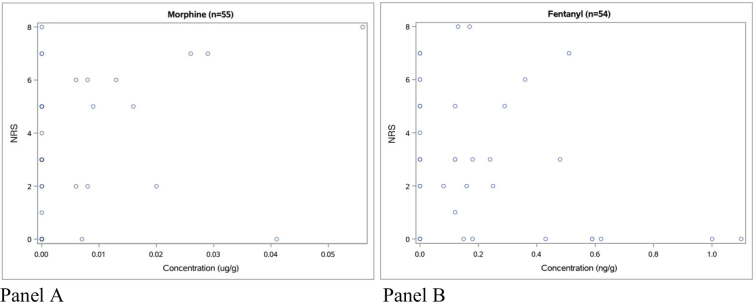
Panel A: morphine concentrations with corresponding NRS value within 4 h of sampling time. Panel B: fentanyl concentrations with corresponding NRS value within 4 h of sampling time.

Analgesic effect evaluated by NRS ratings was not noted as often as RASS values. NRS ratings within 4 h from blood sampling were widely scattered in relation to measured fentanyl and morphine concentrations (Figure 4, panels A and B).

## Discussion

In this prospective single-center study of ICU patients, we found that despite giving standard dosages of sedative and analgesic drugs, there was a wide range of measured blood drug concentrations and corresponding clinical response. Because there were several dose changes in between measured concentrations, and the samples were taken at different time points in relation to the administered dose, this wide range represents both variability in drug exposure within and between patients.

In clinical practice, patients often do not wake up within a reasonable time after sedation is stopped (25). One reason might be that the blood concentrations of sedative and/or analgesic drugs are too high. To some extent, this could be supported by our results, where the two sedatives clonidine and midazolam were found in concentrations above the therapeutic range in 16% (57 of 347) and 17% (38 of 222) of the samples collected during treatment, respectively. Concerning all drugs measured, however, 97% of concentrations were below the upper level in the therapeutic interval. Clonidine was mostly used in addition to other sedatives or in the withdrawal phase of the ICU care.

Since most preexisting reference values in clinical drug concentration monitoring have been analyzed in plasma, it can be difficult to precisely establish whether measured whole blood concentrations were within a therapeutic range. However, for the drugs clonidine and midazolam, which accounted for 78% of the 122 whole blood measurements above therapeutic range, the blood/plasma ratios are less than 1 suggesting even higher concentrations in plasma during the ICU treatment. For dexmedetomidine and ketobemidone, where data on therapeutic concentrations or blood/plasma ratios are missing, it is difficult to say that concentrations did not exceed those expected, and that overdosing was not at hand. However, for dexmedetomidine, population data from the ICU describe concentrations less than 10 μg/L plasma after infusions of 0.1–2.5 μg/kg/h to 21 patients, which is similar to our blood concentrations (26).

Within our cohort, NRS was not documented as often as RASS. This could be because the patients in our ICU are often sedated/intubated and therefore cannot express an NRS rate. In Figure 4, Panel A, the highest morphine concentrations were associated with the highest NRS scores.

Unfortunately, RASS is a scale used only in the ICU setting. Therefore, we did not have the baseline RASS value before ICU admittance. The noted RASS values can be affected by aspects other than sedation given in the ICU such as traumatic brain injury or septic shock.

Like our study, Nies et al. showed a weak correlation between measured midazolam concentrations and RASS values (27). In another study made by Bremer et al., the midazolam concentrations correlated to RASS values, but there was a great interindividual variability, and they concluded that it may be difficult to develop a dosing strategy based on drug concentrations for patients in the ICU (28). Therapeutic drug monitoring on its own might not be the best tool to determine analgesic and sedative drug therapy in the critically ill patient. Analgesia and sedation in ICU patients should be given in accordance with dose intervals and guided by subjective monitoring procedures such as RASS and NRS. To notice, there could be a few patients with drug concentrations above therapeutic interval, with potentially toxic effects, that cannot be discovered by only using dosage guidelines and subjective monitoring procedures.

ICU patients may also have comorbidities with a varying degree of preexisting cardiac, renal, or liver dysfunctions, which might affect the distribution and clearance of the drugs administered (29). The noted 11 occasions of paracetamol plasma concentrations above the therapeutic level of 25 µg/ml (blood/plasma ratio 1.1) can potentially increase the risk for liver toxicity. Any type of organ failure could further enhance the analgesic and sedative effects of the drugs given, but this was outside the scope of this study.

## Strengths and limitations

The strengths of our study are that we were able to investigate a real ICU population, with few exclusion criteria. We could calculate the actual 24-h dosages of the sedatives and analgesic drugs used and note clinical responses corresponding with measured blood concentrations. Since whole blood samples were collected, the study results can directly be used also in forensic postmortem toxicology.

Several limitations must be acknowledged. We measured blood concentrations of sedatives and analgesics only twice daily, at given hours. Measurements were not related to infusion rate changes or boluses given. Any patient’s sedative and/or analgesic drug infusion rate could be adjusted multiple times per day, sometimes several times per hour. Also, procedures were performed in the ICU requiring additional sedation and/or analgesia boluses without the opportunity to measure corresponding drug concentrations. In addition, for drugs with long terminal half-lives, such as clonidine and thiopental, steady state may not have been reached during a short ICU stay.

## Conclusions

In our study population of ICU patients, the standard dosages of analgesic and sedative drugs administered were within the recommended intervals. Despite clinical responses and measured whole blood concentrations showing a wide variation, only 3% of the concentrations were above the therapeutic range. This supports continuous use of standard dosing regimens in combination with clinical evaluation of sedation and pain to tailor analgesia and sedation in the ICU. Further understanding of the complex connection among drug administration, blood concentrations, and corresponding clinical responses in the critically ill patient may be gained by applying pharmacokinetic/pharmacodynamic modeling techniques.

## Data Availability

The dataset used in the present study is available from the authors upon reasonable request. Requests are to be directed to the corresponding author, Ulrica Lennborn, through the e-mail ulrica.lennborn@akademiska.se
